# Alzheimer's disease: are blood and brain markers related? A systematic review

**DOI:** 10.1002/acn3.313

**Published:** 2016-05-11

**Authors:** Ali T. Khan, Richard J. B. Dobson, Martina Sattlecker, Steven J. Kiddle

**Affiliations:** ^1^ GKT School of Medical EducationKing's College LondonLondonUnited Kingdom; ^2^ MRC Social, Genetic and Developmental Psychiatry CentreInstitute of Psychiatry, Psychology and NeuroscienceKing's College LondonLondonUnited Kingdom; ^3^ NIHR Biomedical Research Centre for Mental Health and Biomedical Research Unit for DementiaLondonUnited Kingdom

## Abstract

**Objective:**

Peripheral protein biomarkers of Alzheimer's disease (AD) may help identify novel treatment avenues by allowing early diagnosis, recruitment to clinical trials, and treatment initiation. The purpose of this review was to determine which proteins have been found to be differentially expressed in the AD brain and whether these proteins are also found within the blood of AD patients.

**Methods:**

A two‐stage approach was conducted. The first stage involved conducting a systematic search to identify discovery‐based brain proteomic studies of AD. The second stage involved comparing whether proteins found to be differentially expressed in AD brain were also differentially expressed in the blood.

**Results:**

Across 11 discovery based brain proteomic studies 371 proteins were at different levels in the AD brain. Nine proteins were frequently found, defined as appearing in at least three separate studies. Of these proteins heat‐shock cognate 71 kDa, ubiquitin carboxyl‐terminal hydrolase isozyme L1, and 2′,3′‐cyclic nucleotide 3′ phosphodiesterase alone were found to share a consistent direction of change, being consistently upregulated in studies they appeared in. Eighteen proteins seen as being differentially expressed within the AD brain were present in blood proteomic studies of AD. Only complement C4a was seen multiple times within both the blood and brain proteomic studies.

**Interpretation:**

We report a number of proteins appearing in both the blood and brain of AD patients. Of these proteins, C4a may be a good candidate for further follow‐up in large‐scale replication efforts.

## Introduction

The World Health Organisation (WHO) describes dementia as a syndrome in which memory, thinking, behavior, and the ability to perform everyday activities deteriorate irreversibly. The most common form of dementia is Alzheimer's disease (AD) which presently affects over 35 million individuals globally, and is predicted to affect 115 million people worldwide by 2050.^1^ The total cost of the condition to the UK economy is estimated to be £26.3 billion annually.^2^

At postmortem the brain of AD patients presents with neurodegeneration mostly affecting the medial temporal lobe and neocortical structures.^3^ AD is characterized pathologically by accumulation of amyloid beta (A*β*) peptides, seen as senile plaques, and hyper‐phosphorylated Tau protein apparent as neurofibrillary tangles. The precise triggers for their appearance and propagation remain uncertain. It is therefore unsurprising that management of AD is problematic. A definitive diagnosis of AD can only be made at autopsy when these neuropathological changes are visible. The clinical diagnosis of AD in the living patient is a diagnosis of probability presently. It relies upon a combination of history taking, especially from family members, and either a mental status examination or neuropsychological testing.^4^ Mental status examination involves the condensed mini‐mental state exam and the more thorough Addenbrooke's Cognitive Examination being performed. These examinations suggest the presence of dementia but not the specific type, therefore requiring further investigations. Pharmacological treatment revolves around symptomatic relief. Presently no medication exists that modifies the course of the disease by directly acting on the responsible pathological mechanisms. A number of disease modifying agents are the intense focus of research, although to date many have been found to be ineffective in clinical trials.^5^

Against this background protein biomarkers of AD are assuming increasing importance. A biomarker that corresponds to the early stages of AD would enable the initiation of earlier treatment of this slowly progressing condition, and thus maximize the chances of successful treatment therapy. This could be achieved by identifying patients at an earlier stage of their AD and recruiting them into clinical trials at a point where it is more likely that novel agents will be more effective. The cerebrospinal fluid (CSF) is in direct contact with brain tissue and provides insight into changes experienced by the brain.^6^ A well‐validated finding for the prediction of AD that displays sensitivity and specificity is that the CSF of affected patients sees a reduction in the levels of A*β*_42_, but increased total and phosphorylated Tau levels.^7^ A lumbar puncture, required to extract CSF, is an invasive procedure and thus is far from ideal. Neuroimaging is an alternative method for identifying protein biomarkers. Compounds have been developed that pick up A*β*_42_ deposits in the living brain of sufferers such as the Pittsburgh Compound B, and the FDDNP compound which also binds to Tau proteins.^3^ But their use is expensive and not routine.

Researchers are increasingly turning to peripheral blood protein markers in an attempt to eventually create a simple and cheap blood test that could help clinicians to pick up this disease at the earliest stage possible. The first stage in this process is to identify the AD biomarkers in the blood, and such progress within the field has been recently reviewed.^8^ This review has shown a wide diversity of methods have been applied to this task, but that unfortunately few of the hypothesized biomarkers show cross study replication. However, five proteins were found to have associated with AD‐related phenotypes in five independent studies. Whether and how potential blood biomarkers relate to AD pathology in the brain is largely unknown.

A thorough and systematic analysis of protein levels in brain samples of individuals who had AD will be informative by helping to highlight proteins affected by AD. The proteins whose levels differ most between AD and control brains warrant further research to determine their relationship to pathology and their potential as biomarkers in blood. Furthermore, comparison to the peripheral protein markers already identified will help to determine which peripheral markers are most plausibly related to neurodegeneration. The purpose of this review is to address these issues and to synthesize the information in the hope that future large‐scale replication efforts can be focused on markers which reflect AD pathology both in the blood and brain.

## Methods and Materials

This systematic review comprised two aspects: (1) whether proteins were at different levels in the AD brain relative to control brains; and (2) whether proteins at different levels in AD brains were also at different levels in AD blood samples.

### Differential expression in Alzheimer's disease brain samples

To identify relevant studies a systematic search strategy was conducted in PubMed in December 2014. The phrases “Alzheimer* disease,” “human brain,” and “proteomics” were used and combined with the operative “AND” to return suitable results. To warrant inclusion the study must have been discovery based (i.e., untargeted proteomics) rather than candidate‐based in its approach. This review set out to identify proteins that were at different levels in AD brains as compared to control brains, that is, was the overall level of a protein the same in both AD and control brains, or was it significantly up or downregulated? As such, we limited our findings to proteins displaying this type of change in AD versus control studies. Consequently, studies focusing on specific biochemical modifications occurring to a protein such as oxidation, nitrosylation, and phosphorylation were excluded. In the interests of accuracy and reproducibility of results, only proteins that could be traced back to a specific UniProt ID were included. Finally, to maximize the yield of studies returned from the PubMed search, no specific date range was specified.

### Differential expression in Alzheimer's disease blood samples

A recent systematic review conducted by Chiam et al.^9^ compiled a list of blood protein biomarkers of AD from studies of plasma, serum and leukocyte proteins. Their approach consisted of discovery, rather than candidate, based studies with exceptions made for panel‐based studies including over 100 candidate proteins. This data was further reviewed and amended to remove duplicate protein listings found. Subsequently, proteins identified to be at different levels in AD brain versus control brain samples were compared to this list to ascertain proteins that were also associated with AD‐related phenotypes in blood.

### Statistical analysis

The significance of overlapping protein lists was assessed using a hypergeometric test performed in R.

## Results

### AD brain proteins

**Figure 1:**
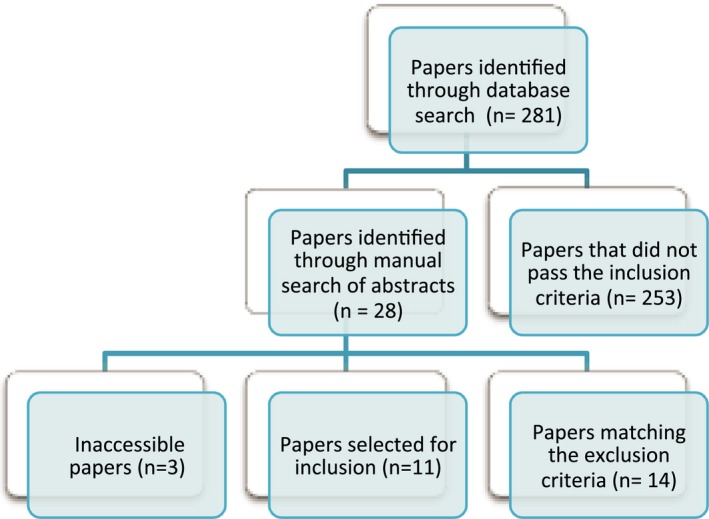
Literature search tree describing the process implemented to source papers for this review. Out of a total of 281 papers identified through database search 11 papers were selected for inclusion.

The initial literature search returned a total of 281 papers matching our search terms. These papers were analyzed through a manual search of their abstracts to identify potentially useful studies. This yielded a total of 28 papers appearing to fulfill the inclusion criteria. The results were then further refined to include only 14 papers, by excluding studies focusing on posttranslational modifications of proteins and studies unable to be identified via a specific UniProt ID. At the time of writing, three papers^10^, ^11^, ^12^ could not be sourced and were not included in the final analysis. Thus the final number of studies selected was 11,^13^, ^14^, ^15^, ^16^, ^17^, ^18^, ^19^, ^20^, ^21^, ^22^, ^23^ with principle brain tissue sites being cortex, hippocampus, and substantia nigra (Table S1). The numbers of papers encountered at each stage of the literature search is displayed in Figure 1.

A total of 371 proteins across 11 studies were found to be at different levels in AD brain samples in at least one study (Table S2). The most frequently found proteins across studies are shown in Table 1. A protein was deemed to be frequently found if it appeared in three or more studies. The most frequently found proteins with a consistent direction of change were heat‐shock cognate 71 kDa protein, ubiquitin carboxyl‐terminal hydrolase isozyme L1, and 2′,3′‐cyclic nucleotide 3′ phosphodiesterase.

**Table 1:** Proteins identified as being the most frequently found proteins from 11 postmortem brain studies of Alzheimer's disease patients

4/11 Studies	3/11 Studies
Creatine kinase B‐type^13^, ^17^, ^18^, ^19^ (Protein downregulated,^13^ Protein upregulated^17^, ^18^, ^19^)	Heat‐shock cognate 71 kDa protein^13^, ^18^, ^20^ (Protein upregulated^13^, ^18^, ^20^)
Glial fibrillary acidic protein^13^, ^19^, ^21^, ^23^ (Protein downregulated,^13^, ^23^ Protein upregulated,^19^, ^23^ Direction not reported^21^)	Ubiquitin carboxyl‐terminal hydrolase isozyme L1^18^, ^19^, ^20^ (Protein upregulated^18^, ^19^, ^20^)
	14‐3‐3 Protein epsilon^13^, ^16^, ^17^ (Protein downregulated,^16^ Protein upregulated^13^, ^17^)
	Dihydropyrimidinase‐related protein^13^, ^20^, ^23^ (Protein downregulated,^20^, ^23^ Protein upregulated^13^)
	Glyceraldehyde‐3‐phosphate dehydrogenase^13^, ^19^, ^20^ (Protein downregulated,^13^, ^19^ Protein upregulated^20^)
	2′,3′‐Cyclic nucleotide 3′ phosphodiesterase^16^, ^20^, ^22^ (Protein upregulated^16^, ^20^, ^22^)
	Alphainternexin^15^, ^19^, ^23^ (Protein downregulated,^19^, ^23^ Protein upregulated^15^)

A protein was deemed to be frequently found if it was present in at least three of the 11 studies.

### AD blood proteins

**Figure 2:**
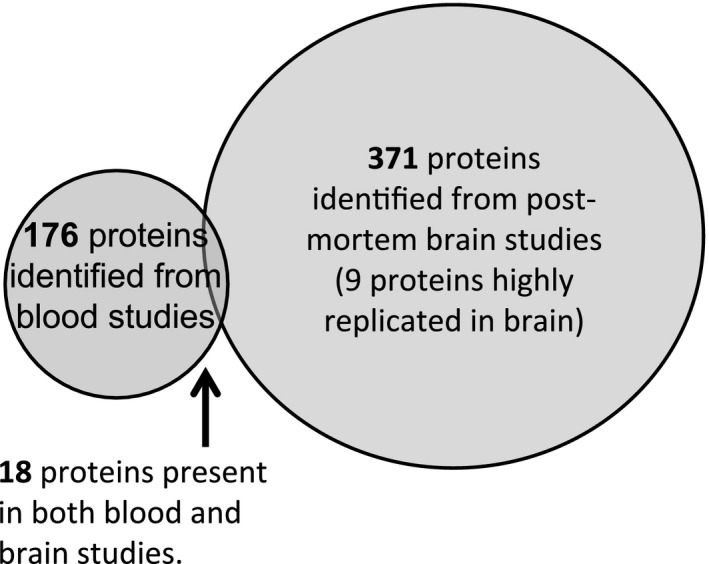
Number of proteins identified in discovery studies associating with Alzheimer's disease (AD) in postmortem brains or AD‐related measures in blood samples. Venn diagram, to approximate scale, is used to demonstrate the overlap. A protein was deemed to be highly replicated if it appeared in 3 or more studies.

Chiam et al. identified 23 papers^25^, ^26^, ^27^, ^28^, ^29^, ^30^, ^31^, ^32^, ^33^, ^34^, ^35^, ^36^, ^37^, ^38^, ^39^, ^40^, ^41^, ^42^, ^43^, ^57^, ^58^, ^59^, ^60^ comprising 18 independent research cohorts and 179 potential blood‐based protein biomarkers.^9^ These results were verified and duplicates found within this study were discarded, giving a total of 176 proteins. The overlap between these 176 proteins and the 371 identified proteins differentially expressed in AD brain amounted to a total of 18 proteins (Fig. 2). The direction of association within the overlap was not considered, this was motivated by the negative association of brain and CSF amyloid beta.^24^

Analysis of the probability of achieving such an overlap by chance alone requires knowledge of the background set, that is, the set of all proteins that could have been detected using the proteomic approaches applied. This is often not known for a specific proteomic approach, and is definitely not known over a wide‐range of different proteomic approaches in different tissues (blood and brain). The overlap was not found to be significant at the 0.05 level (*P* ~ 1) when using a conservative estimate of the size of the background set (*n* = 20,000).

Table S3 displays the 18 proteins shared between both blood and brain studies. Two proteins frequently found within the brain studies, 14‐3‐3 protein epsilon and glyceraldehyde‐3‐phosphate, were found to also be present in the blood studies (Table 2). Only complement C4‐A occurred multiple times in both blood and brain studies, appearing in three blood studies^30^, ^32^, ^36^ and two brain studies.^14^, ^17^

**Table 2:** Proteins identified as being differentially expressed from blood studies of Alzheimer's disease (AD) patients and brain studies of AD patient's

Protein	UniProt ID	Brain studies	Blood studies
14‐3‐3 protein epsilon	P62258	3^13^, ^16^, ^17^	1^43^
Glyceraldehyde‐3‐phosphate dehydrogenase	P04406	3^13^, ^19^, ^20^	1^43^
Calcium/calmodulin‐dependent protein kinase type II subunit alpha	Q9UQM7	2^13^, ^22^	1^40^
Complement C4‐A	P0C0L4	2^14^, ^17^	3^30^, ^32^, ^36^
Gelsolin	P06396	2^17^, ^22^	1^42^

Proteins appearing in at least two brain studies that also appear in at least one blood study are shown. The full list of proteins seen in both types of studies can be seen in Table S3.

## Discussion

The results of our study identify proteins that appear to be associated with AD in both the brain and blood. AD is a complex condition and as such it was perhaps foreseeable that a large number of proteins would be found to be at different levels in AD brain as compared to control brain samples. However, it was encouraging to see a number of brain proteins replicated between studies despite the small sample size and lack of multiple testing corrections of some studies. It was also promising to see proteins appearing in both blood and brain studies.

It is intriguing that there is an agreement, however small, between the two types of studies, that is, some proteins are associated with AD in both the brain and the blood. This is either a sign that some proteins level in multiple tissues (brain and blood) are associated, or could be purely coincidental. Consistent with the possibility that findings are coincidental, the size of the overlap in proteins associated with AD in the brain and blood was not found to be greater than you would expect by chance. However, this analysis is greatly limited by our knowledge of the proteins detectable using these proteomic approaches on samples from these tissues. If instead the proteins level in the blood and brain are truly associated this could be direct – brain levels influencing blood levels or vice versa; or indirect – that their levels are confounded by another factor, for example, AD medication or inflammation. While an indirect link is more likely, the possibility of a direct link needs further consideration.

Ordinarily, the brain is segregated from the vascular system through the blood–brain barrier (BBB). Any exchange of material between the two is minimal and dependent upon specialized transporters. Such an arrangement ensures the brain is well protected from the volatility of the vascular environment. Dysfunction of this important barrier may occur in AD. For example, it has been shown that the BBB begins to breakdown during normal aging starting in the hippocampus, an area of early AD pathology. This breakdown was shown to increase in Mild Cognitive Impairment, which sometimes leads to AD.^44^ Studies have shown blood‐derived toxins to accumulate in postmortem AD,^45^ and in at least a subset of AD patients histological, radiological, and CSF albumin abnormalities have been reported suggesting BBB impairment.^46^ If indeed the BBB is disrupted in AD then this could provide a direct route whereby proteins pass from the brain to the bloodstream (or vice versa) in sufficient quantities as to be detectable. This may explain the association between brain and blood studies that we have found. In this scenario the identities of proteins comparable across studies would assume great importance as they may be involved in AD pathology.

Complement c4a (C4A) was the only protein to associate with AD multiple times in the blood and brain (Table 2). Encouragingly, this protein was upregulated in the cortex of both brain studies it appeared in; reassuringly both of these studies had employed multiple testing corrections (Table S1). C4A is a known marker of inflammation and a feature seen within AD is chronic inflammation, whether occurring primarily as part of the disease or as the body's response to the pathological effects of AD.^47^ This result is therefore in line with current thinking on pathophysiological mechanisms occurring in AD. However, in itself this protein is likely to be fairly non‐specific. Plasma immune markers are affected by different storage methods, concomitant infections and inflammatory illnesses.^48^ Furthermore, the direction of C4A's association within the blood studies was not consistent, with it being reported as downregulated,^32^ upregulated^30^ or unknown^36^ in the three peripheral studies it featured in. It is therefore difficult to distinguish whether this protein is coming from the AD brain, is a non‐specific indicator of AD pathology, or arose as a result of co‐morbidity. As such, in isolation its value as a peripheral biomarker may be limited, but perhaps when used in conjunction with other peripheral markers may be more useful. Indeed, it has previously been suggested that C4A may be used in this way.^32^ Conceivably, a future blood test could act as a prescreening tool to signal the presence of neuropathological changes that can then be investigated further with more specific diagnostic tools such as lumbar punctures or brain scans.

The classical hallmarks of AD brain at postmortem are senile plaques, due to amyloid beta (A*β*) deposition, and neurofibrillary tangles subsequent to tau dysfunction. However, in our review tau is only found to associate with AD in one independent cohort, whereas A*β* was found to associate with AD in just two independent cohorts. While initially surprising, this is likely to be due to the known limitations of the discovery proteomic approaches applied, which may not be capable of accurately quantifying the level of these proteins.

Contrastingly, we report three brain proteins (heat‐shock cognate 71 kDa protein [HSC70], ubiquitin carboxyl‐terminal hydrolase isozyme L1 [UCHL1], and 2′,3′‐cyclic nucleotide 3′ phosphodiesterase [CNPase]) that were consistently upregulated in all brain studies that they appeared in (Table 1). These results should be treated with caution as none of these studies used multiple testing corrections, but the repeated findings do suggest they are worth retesting in larger studies. HSC70 belongs to the heat shock 70 family of proteins that serves to protect neurons from protein aggregation and toxicity. It has recently been suggested that HSC70 slows down the rate of tau clearance in the AD brain^49^ which perhaps explains its consistent upregulation. Previously it had been said that downregulation of HSC70 is responsible for the impaired protein clearance seen in the AD brain.^50^ UCHL1 is a deubiquinating enzyme and over‐expression has been shown to delay AD progression in vivo,^51^ although low levels have been reported in postmortem brain tissue from AD patients.^52^ CNPase plays a role in the synthesis and maintenance of myelin membranes with decreased levels reported in AD.^53^ With this in mind, it is positive that these three brain proteins share a consistent direction of association across our reviewed studies, but there is some discrepancy with regards to previously reported directions (see above). This incongruity may be because of a lack of multiple testing correction in these studies. These proteins were also not confined to a specific area of the brain. For example, HSC70 and CNPase were found within the substantia nigra, cortex and hippocampus sites, whereas UCHL1 was found in the hippocampus and cortex (Table S1).

Creatine‐kinase B type (CKB) is another promising but inconsistent candidate. This was one of the most frequently found proteins within the brain studies. CKB was reported to be upregulated in three studies (importantly one of these three studies did correct for multiple testing) and downregulated in one study (which did not correct for multiple testing). With this in mind it is conceivable that CKB is another protein, that is, consistently upregulated within the AD brain. CKB has a vital role in the cellular energetics of the brain and is highly expressed within the hippocampus, an area of the brain critically affected in AD.^54^ However, it is not known to have a direct link to AD pathology.

A lot of the reviewed findings may simply be coincidental. Strengthening this viewpoint is the observation that very few proteins were seen multiple times as being differentially expressed in AD brain samples, and that directions of association were sometimes inconsistent. This has also been seen in a review of blood protein markers of AD.^8^ Large methodological limitations (e.g., small sample size, and lack of multiple testing corrections) and study differences (e.g., proteomic approaches, postmortem time in processing brain tissue, and physiologic differential expression of proteins at sites) may be the culprit with regards to this issue of replicability. The gold standard of AD diagnosis remains postmortem analysis of the brain and our results are derived from such studies. However, the blood studies reviewed by Kiddle et al.^8^ and Chiam et al.^9^ are obtained from living patients given a clinical diagnosis of AD. This difference in diagnosis between blood and brain studies (i.e., clinical vs. postmortem confirmed) represented a methodological difference that should be considered when interpreting results as there is a possibility that some of the blood results were from patients misdiagnosed as having AD.

It is also disappointing that information about the severity of AD along with comorbid status was not reported within brain studies (Table S1). This is significant as early AD differs from late AD with regards to inflammatory profiles of the brain^55^ and within the studies there is likely heterogeneity of disease severity. Also, the burden of comorbid neurological disorders is higher within the elderly population^56^ with each condition affecting protein expression.^9^ These limitations prevent truer comparisons being made. Moreover, only 3/11 of the studies reviewed used multiple testing corrections to determine differential expression suggesting many hypothesized markers may be false positives. This could be investigated using specific follow‐up experiments.

Finally, we have focused on the blood–brain comparison as a systematic review on blood based protein markers of AD has already been performed by this group.^8^ Furthermore, if a blood protein marker of AD were to be found then this would have a significant impact on the research community. However, an important aspect that needs consideration is whether there is overlap of proteins present within the brain and CSF of AD patients, as the CSF more intimately represents neuronal changes. Future studies reviewing this particular facet will likely further inform the field.

## Conclusion

We have reviewed a large number of proteins whose levels in the brain appear to associate with AD. A few proteins in postmortem brain samples were seen to associate with AD across multiple studies. In addition the level of some of these proteins in blood samples have already been shown to associate with AD‐related phenotypes, suggesting a possible link between their levels in brain and blood in response to AD. However, the robustness of these links has not been sufficiently tested, especially not within a single study. Large‐scale replication efforts are needed to clarify these links and to establish whether proteins found herein are specific to AD.

## Conflict of Interest

None declared.

## Supporting information


**Table S1.** Studies selected for inclusion with information pertaining to sample size, origins of brain tissue, age of participants and proteomic methods employed. * = single numbers are means, two numbers joined by a hyphen are ranges.

**Table S2.** Proteins identified as being expressed within the brain across 11 different studies yielded from a systematic search conducted in the PubMed search engine.

**Table S3.** Proteins identified as being differentially expressed from blood studies of Alzheimer's disease (AD) patients and brain studies of AD patient.
