# Supplementary material for: Alzheimer's disease: are blood and brain markers related? A systematic review
Source: Ann Clin Transl Neurol. 2016 May 11;3(6):455–62. doi: 10.1002/acn3.313 (PMC4891999; doi:10.1002/acn3.313)
Supplement: Supplementary file 1 — Table S1. Studies selected for inclusion with information pertaining to sample size, origins of brain tissue, age of participants and proteomic methods employed. * = single numbers are means, two numbers joined by a hyphen are ranges. [file ACN3-3-455-s001.docx]

Supplementary Table 1 Studies selected for inclusion with information pertaining to sample size, origins of brain tissue, age of participants, proteomic methods and statistical analyses employed. * = single numbers are means, two numbers joined by a hyphen are ranges.

| Study | Tissue sample obtained from | Brain Tissue | Sample size (CTL/AD) | Age range of participants in years (mean or range*), stage of AD, post-mortem interval processing times, and presence/absence of any comorbidities | Proteomic techniques | Statistical analysis | Multiple testing correction |
| --- | --- | --- | --- | --- | --- | --- | --- |
| Zahid et al. [13] | MRC Sudden Death Bank brain, The University of Edinburgh UK | Hippocampus, Substantia Nigra, Cortex | 5/9 | 72  PMI: 24 hour  Well characterised AD, comorbid status unknown | 2DE  ESI-QTOF-MS/MS | \|Log2(FC)\| > 1  and t-test p<0.05. | None |
| Bai et al. [14] | ADRC Brain Bank at Emory University, Rush Alzheimer's Disease Centre's Religious Orders Study at Rush University Medical Centre, the University of Washington ADRC, and the University of Kentucky ADRC | Cortex | 10/10 | AD: 55-81  CTL: 52-75  Well characterised AD, comorbid status unknown | LC-MS/MS of human brain-insoluble proteome | Consensus of (1) G-test (based on chi-squared) and (2) z-test. | (1) P < 0.04 (estimated to give FDR < 0.05) and (2) FDR < 0.05. |
| Zhou et al. [15] | ADRC, CND Brain Bank at Emory University School of Medicine | Cortex | 2/4 ( two AD only ‘possible AD’ ) | AD: 66-76  CTL: 68-75  Matched subjects (± 3 years)  Well characterised AD, comorbid status unknown | LC-MS/MS. Extracted ion current based label-free protein quantification.  Western blot analyses. | \|Log2(FC)\| > 1 (~99% confidence interval) | None |
| Chen et al. [16] | Human Brain and Spinal Fluid Resource Center, VA West Los Angeles Healthcare, Los Angeles, CA 90073 | Substantia nigra | 5/5 as well as 5 HC patients, 5 MS patients | AD: 72-94  HC: 49-65  MS: 47-71  CTL: 58-76  PMI: <30hrs  Well characterised cases, comorbid status unknown | Nano-UPLC-MSE Tandem MS.  Gel Electrophoresis and Western Blotting. | FC >1.2 and t-test p < 0.05 | None |
| Andreev et al. [17] | ADRC brain bank University of Michigan | Cortex | 10/10 | AD: 78 (73–83), Braak staging 5.4, PMI <10 hours, comorbid status unknown  CTL: 76.4±4.8, (69–83); Braak staging 0.3, PMI <24 hours | AMT LC/MS (/MS) Western blotting. | Wilcoxon test. | FDR (q-value < 0.05) |
| Donovan et al. [18] | ADRC brain bank Emory | Frontal Cortex | 5/5 | Cases: 58-71, pathologically confirmed AD Braak staging 5 or 6, comorbid status unknown  Controls: 57-69 | LC-MS/MS immunoblotting | G-test and p value of <0.01 | None. |
| Skorobogatko et al. [19] | No information | Cortical synapses | 1/1 | Age matched control, no age given | iTRAQ in LTQ ion trap mass spectrometry | >30% change from a 1:1 ratio | None |
| Sultana et al. [20] | Rapid Autopsy Program of the University of Kentucky Alzheimer's Disease Centre | Hippocampus | 6/6 | Cases: 84.5 ± 5.2  Control: 85.8 ± 4.1  PMI: average <3hours, AD patients displayed progressive intellectual decline and met NINCDS-ADRDA Workgroup criteria for the clinical diagnosis of probable AD | 2DE | t-test p <0.05 | None |
| Osorio et al. [21] | J. & K. Bryan ADRC | Hippocampus | 1D-GE : 1/2  2D-DIGE : 3/6 | 78 – 85. Matched subjects (± 2 years) | 2DE and imaging  Western blot | 1D-GE : visual inspection.  2D-DIGE : 95% percentile | None |
| Melanson et al. [22] | Biochain Institute (Hayward CA) | Frontal lobe | 1/1 | Case: 58  Control: 82 | Labelling  2D-LC-MS/MS | \|Log2(FC)\| > ~0.48 | None |
| Tsuji et al. [23] | No information | Temporal cortices | 15/15 | Cases: 63-94  Diagnosed with clinical and histopathological AD  Control: 60-87  PMI: <24 hours, | 2DGE  nanoESI  MS/MS Q-TOF | ANOVA | Bonferroni (p < 0.05) |

Abbreviations: 1D-GE = One-dimensional gel electrophoresis; 2DE = Two-dimensional gel electrophoresis; 2D-DIGE = Two-dimensional differential in gel electrophoresis; AD = Alzheimer’s disease; ADRC = Alzheimer's Disease Research Centre; AMT = Accurate mass and time tag; ANOVA = Analysis of Variance; CND = Centre for Neurodegenerative Disease; CTL = Control subject; ESI-QTOF-MS/MS = Electrospray ionization quadropole time of flight tandem MS; FDR = False discovery rate; HD = Huntingtons disease; iTRAQ = Isobaric tag for relative and absolute quantification; LC-MS/MS = Liquid chromatography tandem mass spectrometry; MRC = Medical Research Council; MS = Multiple Sclerosis; MS/MS Q-TOF = tandem mass spectrometry utilising a quadrupole time of flight; nanoESI= nano-electrospray ionisation; PMI = Post mortem interval; UPLC-MSE = Ultra-performance liquid chromatography mass spectrometry elevated energy
