# Supplementary material for: Alzheimer's disease: are blood and brain markers related? A systematic review
Source: Ann Clin Transl Neurol. 2016 May 11;3(6):455–62. doi: 10.1002/acn3.313 (PMC4891999; doi:10.1002/acn3.313)
Supplement: Supplementary file 2 — Table S2. Proteins identified as being expressed within the brain across 11 different studies yielded from a systematic search conducted in the PubMed search engine. [file ACN3-3-455-s002.docx]

Supplementary Table 2 - Proteins identified as being expressed within the brain across 11 different studies yielded from a systematic search conducted in the PubMed search engine

| **Protein name** | **UniProt ID** | **Number of studies in which the brain level of this protein has associated with an AD-related phenotype** | **Zahid et al 2014 ^13^** | **Bai et al 2013 ^14^** | **Zhou et al 2013 ^15^** | **Chen et al 2012 ^16^** | **Andreev et al 2012 ^17^** | **Donovan et al 2012**  **^18^** | **Skorobogatko et al 2011 ^19^** | **Sultana et al 2007 ^20^** | **Osorio et al 2007 ^21^** | **Melanson et al 2006 ^22^** | **Tsuji et al 2002 ^23^** |  |
| --- | --- | --- | --- | --- | --- | --- | --- | --- | --- | --- | --- | --- | --- | --- |
| **Creatine kinase B-type** | **P12277** | **4** | **1** |  |  |  | **1** | **1** | **1** |  |  |  |  |  |
| **Glial fibrillary acidic protein** | **P14136** | **4** | **1** |  |  |  |  |  | **1** |  | **1** |  | **1** |  |
| **Heat shock cognate 71 kDa protein** | **P11142** | **3** | **1** |  |  |  |  | **1** |  | **1** |  |  |  |  |
| **ubiquitin carboxyl-terminal hydrolase isozyme L1** | **P09936** | **3** |  |  |  |  |  | **1** | **1** | **1** |  |  |  |  |
| **14-3-3 protein epsilon** | **P62258** | **3** | **1** |  |  | **1** | **1** |  |  |  |  |  |  |  |
| **Dihydropyrimidinase-related protein** | **Q16555** | **3** | **1** |  |  |  |  |  |  | **1** |  |  | **1** |  |
| **Glyceraldehyde-3-phosphate dehydrogenase** | **P04406** | **3** | **1** |  |  |  |  |  | **1** | **1** |  |  |  |  |
| **2',3'-cyclic nucleotide 3' phosphodiesterase** | **P09543** | **3** |  |  |  | **1** |  |  |  | **1** |  | **1** |  |  |
| **Alpha-internexin** | **Q16352** | **3** |  |  | **1** |  |  |  | **1** |  |  |  | **1** |  |
| **14-3-3 protein beta/alpha** | **P31946** | **2** | **1** |  |  |  | **1** |  |  |  |  |  |  |  |
| **60 kDa heat shock protein, mitochondrial** | **P10809** | **2** | **1** |  |  |  |  |  |  |  |  |  | **1** |  |
| **Alpha-enolase** | **P06733** | **2** | **1** |  |  |  |  |  |  | **1** |  |  |  |  |
| **Annexin A5** | **P08758** | **2** | **1** |  |  |  | **1** |  |  |  |  |  |  |  |
| **Calmodulin** | **P62158** | **2** | **1** |  |  | **1** |  |  |  |  |  |  |  |  |
| **Ferritin heavy chain** | **P02794** | **2** |  |  |  |  | **1** |  |  | **1** |  |  |  |  |
| **HEAT SHOCK 70 kDa protein 2** | **P54652** | **2** |  |  |  | **1** |  |  | **1** |  |  |  |  |  |
| **microtubule-associated protein tau** | **P10636** | **2** |  | **1** |  |  |  | **1** |  |  |  |  |  |  |
| **Phosphoglycerate mutase 1** | **P18669** | **2** |  |  | **1** |  |  |  |  | **1** |  |  |  |  |
| **Pyruvate kinase isozymes M1/M2** | **P14618** | **2** |  |  |  |  | **1** | **1** |  |  |  |  |  |  |
| **succinyl-CoA:3-ketoacid coenzyme A transferase 1, mitochondrial precursor** | **P55809** | **2** |  |  | **1** |  |  |  |  | **1** |  |  |  |  |
| **Synapsin-2** | **Q92777** | **2** | **1** |  |  |  |  |  | **1** |  |  |  |  |  |
| **Syntaxin-binding protein 1** | **P61764** | **2** | **1** |  |  |  |  | **1** |  |  |  |  |  |  |
| **ADP-ribosylation factor 3** | **P61204** | **2** |  |  |  |  | **1** |  |  |  |  | **1** |  |  |
| **Amyloid beta A4 protein** | **P05067** | **2** |  | **1** |  |  | **1** |  |  |  |  |  |  |  |
| **Aspartate aminotransferase, mitochondrial** | **P00505** | **2** | **1** |  |  |  |  | **1** |  |  |  |  |  |  |
| **ATP synthase subunit alpha, mitochondrial** | **P25705** | **2** | **1** |  |  |  |  |  |  |  |  |  | **1** |  |
| **Calcium/calmodulin-dependent protein kinase type II subunit alpha** | **Q9UQM7** | **2** | **1** |  |  |  |  |  |  |  |  | **1** |  |  |
| **Calpain-2 catalytic subunit** | **P17655** | **2** |  |  |  |  | **1** |  |  |  |  |  | **1** |  |
| **Carbonic anhydrase 2** | **P00918** | **2** |  |  |  |  |  |  |  | **1** |  | **1** |  |  |
| **Complement C4-A** | **P0C0L4** | **2** |  | **1** |  |  | **1** |  |  |  |  |  |  |  |
| **Exportin-7** | **Q9UIA9** | **2** |  | **1** |  |  | **1** |  |  |  |  |  |  |  |
| **Fascin** | **Q16658** | **2** | **1** |  | **1** |  |  |  |  |  |  |  |  |  |
| **Fumarate hydratase** | **P07954** | **2** | **1** | **1** |  |  |  |  |  |  |  |  |  |  |
| **Gelsolin** | **P06396** | **2** |  |  |  |  | **1** |  |  |  |  | **1** |  |  |
| **Homer protein homolog 1** | **Q86YM7** | **2** |  |  | **1** |  | **1** |  |  |  |  |  |  |  |
| **Myelin basic protein** | **P02686** | **2** |  |  |  |  |  |  |  |  | **1** | **1** |  |  |
| **Myotrophin** | **P58546** | **2** |  |  |  |  | **1** |  |  |  |  | **1** |  |  |
| **NAD dependent deacetylase sirturin 2** | **Q8IXJ6** | **2** |  |  |  | **1** |  |  |  |  |  | **1** |  |  |
| **NADH dehydrogenase [ubiquinone] 1 alpha subcomplex subunit 9, mitochondrial** | **Q16795** | **2** |  |  |  |  | **1** |  |  |  |  | **1** |  |  |
| **NADH dehydrogenase [ubiquinone] flavoprotein 2, mitochondrial** | **P19404** | **2** |  |  | **1** |  |  |  |  |  |  | **1** |  |  |
| **NAD(P) transhydrogenase, mitochondrial** | **Q13423** | **2** |  | **1** |  |  |  |  |  |  |  | **1** |  |  |
| **Protein-arginine deiminase type-2** | **Q9Y2J8** | **2** |  |  |  | **1** | **1** |  |  |  |  |  |  |  |
| **Protein S100-A1** | **P23297** | **2** |  |  |  | **1** | **1** |  |  |  |  |  |  |  |
| **versican core protein** | **P13611** | **2** |  |  |  |  |  | **1** |  |  |  | **1** |  |  |
| **Voltage-dependent anion-selective channel protein 1** | **P21796** | **2** | **1** |  |  |  |  |  |  |  |  | **1** |  |  |
| **Calcium/calmodulin-dependent protein kinase type II subunit delta** | **Q13557** | **2** | **1** |  |  |  |  |  |  |  |  | **1** |  |  |
| **Aconitate hydratase, mitochondrial** | **Q99798** | **1** | **1** |  |  |  |  |  |  |  |  |  |  |  |
| **Glycogen phosphorylase, brain form** | **P11216** | **1** |  |  |  |  | **1** |  |  |  |  |  |  |  |
| **Actin, cytoplasmic 1** | **P60709** | **1** | **1** |  |  |  |  |  |  |  |  |  |  |  |
| **Peroxiredoxin-2** | **P32119** | **1** |  |  |  |  |  |  |  | **1** |  |  |  |  |
| **Peroxiredoxin-6** | **P30041** | **1** |  |  |  |  | **1** |  |  |  |  |  |  |  |
| **Thioredoxin-dependent peroxide reductase, mitochondrial** | **P30048** | **1** |  |  | **1** |  |  |  |  |  |  |  |  |  |
| **Triose phosphate isomerase** | **P60174** | **1** |  |  |  |  |  |  |  | **1** |  |  |  |  |
| **4-aminobutyrate aminotransferase, mitochondrial** | **P80404** | **1** |  | **1** |  |  |  |  |  |  |  |  |  |  |
| **78 kDa glucose-regulated protein** | **P11021** | **1** |  |  |  | **1** |  |  |  |  |  |  |  |  |
| **14-3-3 protein theta** | **P27348** | **1** |  |  |  | **1** |  |  |  |  |  |  |  |  |
| **adenylate kinase isoenzyme 1** | **P00568** | **1** |  |  |  |  |  |  |  | **1** |  |  |  |  |
| **Aldehyde dehydrogenase, mitochondrial** | **P05091** | **1** |  |  |  |  |  |  | **1** |  |  |  |  |  |
| **Amphiphysin** | **P49418** | **1** |  | **1** |  |  |  |  |  |  |  |  |  |  |
| **Apolipoprotein E** | **P02649** | **1** |  | **1** |  |  |  |  |  |  |  |  |  |  |
| **ATP synthase subunit beta, mitochondrial** | **P06576** | **1** |  |  |  |  |  |  |  |  |  |  | **1** |  |
| **ATP synthase subunit d, mitochondrial** | **O75947** | **1** | **1** |  |  |  |  |  |  |  |  |  |  |  |
| **Beta-enolase** | **P13929** | **1** |  |  |  |  |  |  |  |  |  |  | **1** |  |
| **brain acid soluble protein 1** | **P80723** | **1** |  |  | **1** |  |  |  |  |  |  |  |  |  |
| **Carbonyl reductase [NADPH] 1** | **P16152** | **1** | **1** |  |  |  |  |  |  |  |  |  |  |  |
| **Creatine kinase U-type, mitochondrial** | **P12532** | **1** | **1** |  |  |  |  |  |  |  |  |  |  |  |
| **D-3-phosphoglycerate dehydrogenase** | **O43175** | **1** |  |  | **1** |  |  |  |  |  |  |  |  |  |
| **Dihydrolipoyl dehydrogenase, mitochondrial** | **P09622** | **1** | **1** |  |  |  |  |  |  |  |  |  |  |  |
| **Dynamin-1** | **Q05193** | **1** | **1** |  |  |  |  |  |  |  |  |  |  |  |
| **Fatty acid-binding protein, epidermal** | **Q01469** | **1** |  |  |  |  | **1** |  |  |  |  |  |  |  |
| **Fatty acid-binding protein, heart** | **P05413** | **1** |  |  |  |  |  |  |  |  |  |  | **1** |  |
| **Fructose-bisphosphate aldolase A** | **P04075** | **1** | **1** |  |  |  |  |  |  |  |  |  |  |  |
| **Galectin-1** | **P09382** | **1** |  |  |  |  |  |  |  |  |  | **1** |  |  |
| **Guanine deaminase** | **Q9Y2T3** | **1** | **1** |  |  |  |  |  |  |  |  |  |  |  |
| **Guanine nucleotide-binding protein G(I)/G(S)/G(T) subunit beta-1** | **P62873** | **1** |  |  |  |  |  |  |  |  |  |  | **1** |  |
| **Heat shock protein beta-1** | **P04792** | **1** |  |  |  |  | **1** |  |  |  |  |  |  |  |
| **heat shock protein HSP 90-beta** | **P08238** | **1** |  |  |  |  |  | **1** |  |  |  |  |  |  |
| **Heat shock 70 kDa protein 12A** | **O43301** | **1** |  |  |  |  | **1** |  |  |  |  |  |  |  |
| **Heat shock protein 75 kDa, mitochondrial** | **Q12931** | **1** |  |  |  |  |  |  | **1** |  |  |  |  |  |
| **Hemoglobin subunit alpha** | **P69905** | **1** | **1** |  |  |  |  |  |  |  |  |  |  |  |
| **Histidine triad nucleotide-binding protein 1** | **P49773** | **1** |  |  |  |  | **1** |  |  |  |  |  |  |  |
| **Isocitrate dehydrogenase [NADP], mitochondrial** | **P48735** | **1** | **1** |  |  |  |  |  |  |  |  |  |  |  |
| **L-lactate dehydrogenase B chain** | **P07195** | **1** | **1** |  |  |  |  |  |  |  |  |  |  |  |
| **Malate dehydrogenase, cytoplasmic** | **P40925** | **1** | **1** |  |  |  |  |  |  |  |  |  |  |  |
| **Malate dehydrogenase, mitochondrial** | **P40926** | **1** |  |  |  |  |  |  | **1** |  |  |  |  |  |
| **Microtubule-associated protein 1B** | **P48621** | **1** |  |  |  |  |  |  | **1** |  |  |  |  |  |
| **Myelin proteolipid protein** | **P60201** | **1** |  |  | **1** |  |  |  |  |  |  |  |  |  |
| **Neurofilament light polypeptide** | **P07196** | **1** |  |  |  |  | **1** |  |  |  |  |  |  |  |
| **Neurofilament medium polypeptide** | **P07197** | **1** |  |  | **1** |  |  |  |  |  |  |  |  |  |
| **Nucleoside diphosphate kinase B** | **P22392** | **1** | **1** |  |  |  |  |  |  |  |  |  |  |  |
| **Peroxiredoxin-1** | **Q06830** | **1** |  |  |  |  | **1** |  |  |  |  |  |  |  |
| **Phosphatidylethanolamine-binding protein 1** | **P30086** | **1** |  |  |  |  | **1** |  |  |  |  |  |  |  |
| **Plasma membrane calcium-transporting ATPase 3** | **Q16720** | **1** |  |  |  |  | **1** |  |  |  |  |  |  |  |
| **protein NDRG2** | **Q9UN36** | **1** |  |  |  | **1** |  |  |  |  |  |  |  |  |
| **Rab GDP dissociation inhibitor alpha** | **P31150** | **1** |  |  |  |  | **1** |  |  |  |  |  |  |  |
| **ras-related GTP-binding protein RAB10** | **P61026** | **1** |  |  | **1** |  |  |  |  |  |  |  |  |  |
| **Ras-related protein Rab-15** | **P59190** | **1** |  |  | **1** |  |  |  |  |  |  |  |  |  |
| **Receptor-type tyrosine-protein phosphatase zeta** | **P23471** | **1** |  | **1** |  |  |  |  |  |  |  |  |  |  |
| **Septin-7** | **Q16181** | **1** | **1** |  |  |  |  |  |  |  |  |  |  |  |
| **Spectrin alpha chain, non-erythrocytic 1** | **Q13813** | **1** |  |  | **1** |  |  |  |  |  |  |  |  |  |
| **Spectrin beta chain, non-erythrocytic 1** | **Q01082** | **1** |  |  | **1** |  |  |  |  |  |  |  |  |  |
| **Stress-70 protein, mitochondrial** | **P38646** | **1** |  |  |  |  |  |  |  |  | **1** |  |  |  |
| **Synaptojanin 1** | **O43426** | **1** |  | **1** |  |  |  |  |  |  |  |  |  |  |
| **Synaptosomal-associated protein 25** | **P60880** | **1** | **1** |  |  |  |  |  |  |  |  |  |  |  |
| **tenascin-R** | **Q92752** | **1** |  |  |  |  |  | **1** |  |  |  |  |  |  |
| **Tubulin beta-4A chain** | **P04350** | **1** |  |  |  |  |  |  |  | **1** |  |  |  |  |
| **Vesicle-fusing ATPase** | **P46459** | **1** | **1** |  |  |  |  |  |  |  |  |  |  |  |
| **V-type proton ATPase subunit B, brain isoform** | **P21281** | **1** |  |  |  |  | **1** |  |  |  |  |  |  |  |
| **Visinin-like protein 1** | **P62760** | **1** |  |  | **1** |  |  |  |  |  |  |  |  |  |
| **[3-methyl-2-oxobutanoate dehydrogenase [lipoamide]] kinase, mitochondrial** | **O14874** | **1** |  |  |  |  | **1** |  |  |  |  |  |  |  |
| **3-hydroxyisobutyrate dehydrogenase, mitochondrial** | **P31937** | **1** |  |  |  |  | **1** |  |  |  |  |  |  |  |
| **5'-AMP-activated protein kinase subunit gamma-1** | **P54619** | **1** |  |  |  |  | **1** |  |  |  |  |  |  |  |
| **26S proteasome non-ATPase regulatory subunit 13** | **Q9UNM6** | **1** |  |  |  |  | **1** |  |  |  |  |  |  |  |
| **26S protease regulatory subunit 6B** | **P43686** | **1** |  |  |  |  | **1** |  |  |  |  |  |  |  |
| **40S ribosomal protein S28** | **P62857** | **1** |  |  |  |  | **1** |  |  |  |  |  |  |  |
| **40S ribosomal protein S13** | **P62277** | **1** |  |  |  |  | **1** |  |  |  |  |  |  |  |
| **40S ribosomal protein S3** | **P23396** | **1** |  |  |  |  | **1** |  |  |  |  |  |  |  |
| **40S ribosomal protein S4, X isoform** | **P62701** | **1** |  |  |  |  | **1** |  |  |  |  |  |  |  |
| **60S ribosomal protein L7** | **P18124** | **1** |  |  |  |  | **1** |  |  |  |  |  |  |  |
| **Actin, cytoplasmic 2** | **Q96E67** | **1** |  |  |  |  |  |  |  |  | **1** |  |  |  |
| **Activated RNA polymerase II transcriptional coactivator p15** | **P53999** | **1** |  |  |  |  | **1** |  |  |  |  |  |  |  |
| **Acyl-coenzyme A thioesterase 9, mitochondrial** | **Q9Y305** | **1** |  |  |  |  | **1** |  |  |  |  |  |  |  |
| **Adenylyl cyclase-associated protein 1** | **Q01518** | **1** |  |  |  |  | **1** |  |  |  |  |  |  |  |
| **Adenylyl cyclase-associated protein 2** | **P40123** | **1** |  |  |  |  | **1** |  |  |  |  |  |  |  |
| **Adipocyte plasma membrane-associated protein** | **Q9HDC9** | **1** |  |  |  |  | **1** |  |  |  |  |  |  |  |
| **ADP-ribosylation factor 5** | **P84085** | **1** |  |  |  |  |  |  |  |  |  | **1** |  |  |
| **AFG3-like protein 2** | **Q9Y4W6** | **1** |  |  |  |  | **1** |  |  |  |  |  |  |  |
| **ATP-dependent DNA helicase 2 subunit 2** | **P13010** | **1** |  |  |  |  | **1** |  |  |  |  |  |  |  |
| **Alpha-1,3/1,6-mannosyltransferase ALG2** | **Q9H553** | **1** |  | **1** |  |  |  |  |  |  |  |  |  |  |
| **alpha-2-macroglobulin** | **P01023** | **1** |  |  |  |  |  | **1** |  |  |  |  |  |  |
| **Alpha-crystallin B chain** | **P02511** | **1** |  |  |  |  |  |  |  |  |  | **1** |  |  |
| **Ankyrin repeat and BTB (POZ) domain containing 1** | **Q969K4** | **1** |  |  |  |  |  |  | **1** |  |  |  |  |  |
| **Annexin A1** | **P04083** | **1** |  |  |  |  | **1** |  |  |  |  |  |  |  |
| **Annexin A6** | **P08133** | **1** |  |  |  |  |  |  |  |  |  | **1** |  |  |
| **Apolipoprotein O** | **Q9BUR5** | **1** |  |  |  |  | **1** |  |  |  |  |  |  |  |
| **Aquaporin-4** | **P55087** | **1** |  |  |  |  | **1** |  |  |  |  |  |  |  |
| **ATF71P** | **Q6VMQ6** | **1** |  |  |  |  |  |  | **1** |  |  |  |  |  |
| **ATPase inhibitor, mitochondrial** | **Q9UII2** | **1** |  |  |  |  | **1** |  |  |  |  |  |  |  |
| **ATP-binding cassette sub-family A member 13** | **Q86UQ4** | **1** |  |  |  |  | **1** |  |  |  |  |  |  |  |
| **ATP synthase subunit b** | **P24539** | **1** |  |  |  | **1** |  |  |  |  |  |  |  |  |
| **ATP synthase subunit gamma, mitochondrial** | **P36542** | **1** | **1** |  |  |  |  |  |  |  |  |  |  |  |
| **B-cell receptor-associated protein 29** | **Q9UHQ4** | **1** |  |  |  |  | **1** |  |  |  |  |  |  |  |
| **Basic immunoglobulin-like variable motif-containing protein** | **Q86UB2** | **1** |  |  |  |  | **1** |  |  |  |  |  |  |  |
| **Beta-centractin** | **P42025** | **1** |  |  | **1** |  |  |  |  |  |  |  |  |  |
| **Beta-tubulin** | **P07437** | **1** |  |  |  |  |  |  | **1** |  |  |  |  |  |
| **Bifunctional aminoacyl-tRNA synthetase** | **P07814** | **1** |  |  |  |  | **1** |  |  |  |  |  |  |  |
| **Cadherin-23** | **Q9H251** | **1** |  |  |  |  | **1** |  |  |  |  |  |  |  |
| **Calbindin** | **P05937** | **1** | **1** |  |  |  |  |  |  |  |  |  |  |  |
| **Calcium/calmodulin-dependent protein kinase type II subunit gamma** | **Q13555** | **1** |  |  |  |  |  |  |  |  |  | **1** |  |  |
| **Calcium signal-modulating cyclophilin ligand** | **P49069** | **1** |  |  |  |  | **1** |  |  |  |  |  |  |  |
| **Carboxypeptidase E** | **P16870** | **1** |  |  |  |  | **1** |  |  |  |  |  |  |  |
| **Caspase-5** | **P51878** | **1** |  |  |  | **1** |  |  |  |  |  |  |  |  |
| **CD9 antigen** | **P21926** | **1** |  |  |  |  |  |  |  |  |  | **1** |  |  |
| **cAMP-dependent protein kinase (PKA) catalytic subunit beta** | **P22694** | **1** |  |  |  |  |  |  | **1** |  |  |  |  |  |
| **cGMP-dependent 3',5'-cyclic phosphodiesterase** | **O00408** | **1** |  |  |  |  | **1** |  |  |  |  |  |  |  |
| **Cell cycle checkpoint protein RAD17** |  | **1** |  |  |  |  | **1** |  |  |  |  |  |  |  |
| **cell division control protein 42 homolog** | **P60953** | **1** |  |  |  | **1** |  |  |  |  |  |  |  |  |
| **Cellular retinoic acid binding protein 1** | **P29762** | **1** |  | **1** |  |  |  |  |  |  |  |  |  |  |
| **Centrin-2** | **P41208** | **1** |  |  |  |  | **1** |  |  |  |  |  |  |  |
| **Chaperonin containing TCP1 (CCT), subunit 7** | **Q99832** | **1** |  |  |  |  |  |  | **1** |  |  |  |  |  |
| **chaperonin containing TCP1, subunit 8 (theta)** | **P50990** | **1** |  |  | **1** |  |  |  |  |  |  |  |  |  |
| **Chloride intracellular channel protein 4** | **Q9Y696** | **1** |  |  |  |  | **1** |  |  |  |  |  |  |  |
| **Choline-phosphate cytidylyltransferase A** | **P49585** | **1** |  |  |  |  | **1** |  |  |  |  |  |  |  |
| **Coiled-coil domain-containing protein 11** | **Q96M91** | **1** |  |  |  |  | **1** |  |  |  |  |  |  |  |
| **Collagen, type XXV, alpha 1** | **Q9BXS0** | **1** |  | **1** |  |  |  |  |  |  |  |  |  |  |
| **COMM domain-containing protein 2** | **Q86X83** | **1** |  |  |  |  | **1** |  |  |  |  |  |  |  |
| **Complement component 1 Q subcomponent-binding protein, mitochondrial** | **Q07021** | **1** |  |  |  |  | **1** |  |  |  |  |  |  |  |
| **Complement component 3** | **P01024** | **1** |  | **1** |  |  |  |  |  |  |  |  |  |  |
| **Complement C4-B** | **P0C0L5** | **1** |  | **1** |  |  |  |  |  |  |  |  |  |  |
| **Coronin-1C** | **Q9ULV4** | **1** |  |  |  |  | **1** |  |  |  |  |  |  |  |
| **Crk-like protein** | **P46109** | **1** |  |  |  |  | **1** |  |  |  |  |  |  |  |
| **Cyclin G-associated kinase** | **O14976** | **1** |  | **1** |  |  |  |  |  |  |  |  |  |  |
| **Cytochrome b-c1 complex subunit Rieske, mitochondrial** | **P47985** | **1** | **1** |  |  |  |  |  |  |  |  |  |  |  |
| **Cytochrome b-c1 complex subunit 1, mitochondrial** | **P31930** | **1** |  |  |  |  |  |  |  |  |  | **1** |  |  |
| **Cytochrome b-c1 complex subunit 2, mitochondrial** | **P22695** | **1** |  |  |  |  |  |  |  |  |  | **1** |  |  |
| **Cytochrome b-c1 complex subunit 6, mitochondrial** | **P07919** | **1** |  |  |  |  |  |  |  |  |  | **1** |  |  |
| **Cytochrome b-c1 complex subunit 7** | **P14927** | **1** |  |  |  |  |  |  |  |  |  | **1** |  |  |
| **Cytochrome b-c1 complex subunit 10** | **O14957** | **1** |  |  |  |  | **1** |  |  |  |  |  |  |  |
| **Cytochrome c oxidase subunit 5B, mitochondrial** | **P10606** | **1** |  |  | **1** |  |  |  |  |  |  |  |  |  |
| **Cytochrome c oxidase subunit 7A2, mitochondrial** | **P14406** | **1** |  |  |  |  |  |  |  |  |  | **1** |  |  |
| **Cytoplasmic FMR1-interacting protein 1** | **Q7L576** | **1** |  |  |  |  | **1** |  |  |  |  |  |  |  |
| **Cytoplasmic dynein 1 light intermediate chain 2** | **O43237** | **1** |  |  |  |  | **1** |  |  |  |  |  |  |  |
| **Cytosolic 10-formyltetrahydrofolate dehydrogenase** | **O75891** | **1** |  | **1** |  |  |  |  |  |  |  |  |  |  |
| **DDRGK domain-containing protein 1** | **Q96HY6** | **1** |  |  |  |  | **1** |  |  |  |  |  |  |  |
| **Delta-aminolevulinic acid dehydratase** | **P13716** | **1** |  |  |  |  | **1** |  |  |  |  |  |  |  |
| **Dipeptidyl-peptidase 2** | **Q9UHL4** | **1** |  |  |  |  | **1** |  |  |  |  |  |  |  |
| **dynactin subunit 2** | **Q13561** | **1** |  |  | **1** |  |  |  |  |  |  |  |  |  |
| **Dynamin-3** | **Q9UQ16** | **1** |  |  |  |  |  |  |  |  |  | **1** |  |  |
| **Dynein light chain Tctex-type 1** | **P63172** | **1** |  |  |  |  | **1** |  |  |  |  |  |  |  |
| **Dystrobrevin alpha** | **Q9Y4J8** | **1** |  | **1** |  |  |  |  |  |  |  |  |  |  |
| **DNA-dependent protein kinase catalytic subunit** | **P78527** | **1** |  |  |  |  | **1** |  |  |  |  |  |  |  |
| **E3 ubiquitin-protein ligase parkin** | **O60260** | **1** |  |  |  |  | **1** |  |  |  |  |  |  |  |
| **EF-hand domain-containing protein D2** | **Q96C19** | **1** |  |  |  |  | **1** |  |  |  |  |  |  |  |
| **Ectonucleotide pyrophosphatase/phosphodiesterase family member 6** | **Q6UWR7** | **1** |  |  |  |  | **1** |  |  |  |  |  |  |  |
| **ELAV-like protein 2** | **Q12926** | **1** |  |  |  |  | **1** |  |  |  |  |  |  |  |
| **Elongation factor Tu, mitochondrial** | **P49411** | **1** | **1** |  |  |  |  |  |  |  |  |  |  |  |
| **Endoplasmic reticulum protein ERp29** | **P30040** | **1** |  |  |  |  | **1** |  |  |  |  |  |  |  |
| **Epimerase family protein SDR39U1** | **Q9NRG7** | **1** |  |  |  |  | **1** |  |  |  |  |  |  |  |
| **ERO1-like protein alpha** | **Q96HE7** | **1** |  |  |  |  | **1** |  |  |  |  |  |  |  |
| **ES1 protein homolog, mitochondrial** | **P30042** | **1** |  |  | **1** |  |  |  |  |  |  |  |  |  |
| **Eukaryotic translation initiation factor 3 subunit M** | **Q7L2H7** | **1** |  |  |  |  | **1** |  |  |  |  |  |  |  |
| **Eukaryotic translation initiation factor 4 gamma 2** | **P78344** | **1** |  |  |  |  | **1** |  |  |  |  |  |  |  |
| **Eukaryotic translation initiation factor 4H** | **Q15056** | **1** |  |  |  |  | **1** |  |  |  |  |  |  |  |
| **Eukaryotic translation initiation factor 3 subunit F** | **O00303** | **1** |  |  |  |  | **1** |  |  |  |  |  |  |  |
| **Excitatory amino acid transporter 2** | **P43004** | **1** |  |  |  |  |  |  |  |  |  | **1** |  |  |
| **F-box-like/WD repeat-containing protein TBL1Y** | **Q9BQ87** | **1** |  |  |  |  | **1** |  |  |  |  |  |  |  |
| **FAS-associated factor 1** | **Q9UNN5** | **1** |  |  |  |  | **1** |  |  |  |  |  |  |  |
| **Fas apoptotic inhibitory molecule 2** | **Q9BWQ8** | **1** |  |  |  |  | **1** |  |  |  |  |  |  |  |
| **Fructose-bisphosphate aldolase** | **H3BQN4** | **1** |  |  |  |  |  |  |  | **1** |  |  |  |  |
| **FXYD domain-containing ion transport regulator 7** | **P58549** | **1** |  |  |  |  | **1** |  |  |  |  |  |  |  |
| **G-rich sequence factor 1** | **Q12849** | **1** |  |  |  |  | **1** |  |  |  |  |  |  |  |
| **Gamma-soluble NSF attachment protein** | **Q99747** | **1** |  |  |  |  | **1** |  |  |  |  |  |  |  |
| **Ganglioside-induced differentiation-associated protein 1** | **Q8TB36** | **1** |  |  |  |  | **1** |  |  |  |  |  |  |  |
| **Glia-activating factor** | **P31371** | **1** |  |  |  | **1** |  |  |  |  |  |  |  |  |
| **glucose-6-phosphate isomerase** | **P06744** | **1** |  |  | **1** |  |  |  |  |  |  |  |  |  |
| **Glutamate receptor interacting protein 1** | **Q9Y3R0** | **1** |  | **1** |  |  |  |  |  |  |  |  |  |  |
| **Glutaminase** | **O94925** | **1** |  |  |  |  |  |  | **1** |  |  |  |  |  |
| **Glutathione reductase, mitochondrial** | **P00390** | **1** |  |  |  |  | **1** |  |  |  |  |  |  |  |
| **Glutathione S-transferase Mu 3** | **P21266** | **1** |  |  |  |  |  |  |  |  |  | **1** |  |  |
| **Glycerol-3-phosphate dehydrogenase [NAD+], cytoplasmic** |  | **1** |  |  |  |  | **1** |  |  |  |  |  |  |  |
| **GrpE protein homolog 1, mitochondrial** | **Q9HAV7** | **1** |  |  |  |  | **1** |  |  |  |  |  |  |  |
| **GTPase-activating protein and VPS9 domain-containing protein 1** | **Q14C86** | **1** |  | **1** |  |  |  |  |  |  |  |  |  |  |
| **Guanine nucleotide-binding protein G(o) subunit alpha** | **P09471** | **1** |  |  |  |  |  |  | **1** |  |  |  |  |  |
| **Heat shock factor-binding protein 1** | **O75506** | **1** |  |  |  |  | **1** |  |  |  |  |  |  |  |
| **Hepatoma-derived growth factor-related protein 3** | **Q9Y3E1** | **1** |  |  |  |  | **1** |  |  |  |  |  |  |  |
| **Heterogeneous nuclear ribonucleoprotein A3** | **P51991** | **1** |  |  |  |  | **1** |  |  |  |  |  |  |  |
| **Hexokinase-1** | **P19367** | **1** |  |  |  |  |  |  |  |  |  | **1** |  |  |
| **Histone H4** | **P62805** | **1** |  |  |  |  | **1** |  |  |  |  |  |  |  |
| **Huntingtin-interacting protein 1-related protein** | **O75146** | **1** |  |  |  |  | **1** |  |  |  |  |  |  |  |
| **Ig alpha-1 chain C region** | **P01876** | **1** | **1** |  |  |  |  |  |  |  |  |  |  |  |
| **Ig gamma-1 chain C region** | **P01857** | **1** | **1** |  |  |  |  |  |  |  |  |  |  |  |
| **Immunoglobulin-like and fibronectin type III domain-containing protein 1** | **Q86VF2** | **1** |  |  |  |  | **1** |  |  |  |  |  |  |  |
| **Inverted formin-2** | **Q27J81** | **1** |  |  |  |  | **1** |  |  |  |  |  |  |  |
| **Integrin beta-6** | **P18564** | **1** |  |  |  |  | **1** |  |  |  |  |  |  |  |
| **Isochorismatase domain-containing protein 1** | **Q96CN7** | **1** |  |  |  |  | **1** |  |  |  |  |  |  |  |
| **Isovaleryl-CoA dehydrogenase, mitochondrial** | **P26440** | **1** |  |  |  |  | **1** |  |  |  |  |  |  |  |
| **Lactoylglutathione lyase** | **Q04760** | **1** | **1** |  |  |  |  |  |  |  |  |  |  |  |
| **LanC-like protein 1** | **O43813** | **1** | **1** |  |  |  |  |  |  |  |  |  |  |  |
| **L-lactate dehydrogenase** | **B7Z5E3** | **1** |  |  |  | **1** |  |  |  |  |  |  |  |  |
| **Lamin-B1** | **P20700** | **1** |  |  |  |  | **1** |  |  |  |  |  |  |  |
| **Large proline-rich protein BAT3** | **P46379** | **1** |  |  |  |  | **1** |  |  |  |  |  |  |  |
| **Lymphoid-restricted membrane protein** | **Q12912** | **1** |  |  |  |  | **1** |  |  |  |  |  |  |  |
| **Lysosome-associated membrane glycoprotein 1** | **P11279** | **1** |  |  |  |  | **1** |  |  |  |  |  |  |  |
| **Metabotropic glutamate receptor 2** | **Q14416** | **1** |  |  |  |  | **1** |  |  |  |  |  |  |  |
| **Methionine adenosyltransferase 2 subunit beta** | **Q9NZL9** | **1** |  |  |  |  | **1** |  |  |  |  |  |  |  |
| **MICOS complex subunit MIC60** | **Q16891** | **1** |  |  | **1** |  |  |  |  |  |  |  |  |  |
| **Microtubule-associated protein 1S** | **Q66K74** | **1** |  |  |  |  | **1** |  |  |  |  |  |  |  |
| **Microtubule-associated protein RP/EB family member 1** | **Q15691** | **1** |  |  |  |  | **1** |  |  |  |  |  |  |  |
| **Mitochondrial fission regulator 1** | **Q15390** | **1** |  |  |  |  | **1** |  |  |  |  |  |  |  |
| **mitochondrial carrier homolog 2** | **Q9Y6C9** | **1** |  |  | **1** |  |  |  |  |  |  |  |  |  |
| **Mitochondrial glutamate carrier 1** | **Q9H936** | **1** |  |  |  |  | **1** |  |  |  |  |  |  |  |
| **Mitochondrial-processing peptidase subunit beta** | **O75439** | **1** |  | **1** |  |  |  |  |  |  |  |  |  |  |
| **Myomegalin** | **Q5VU43** | **1** |  |  |  |  | **1** |  |  |  |  |  |  |  |
| **Myelin-oligodendrocyte glycoprotein** | **Q16653** | **1** |  |  |  |  |  |  |  |  |  | **1** |  |  |
| **Myosin-15** | **Q9Y2K3** | **1** |  |  |  |  | **1** |  |  |  |  |  |  |  |
| **Myristoylated alanine-rich C-kinase substrate** | **P29966** | **1** |  |  |  |  |  |  |  |  |  | **1** |  |  |
| **N-acetyl-D-glucosamine kinase** | **Q9UJ70** | **1** |  |  |  |  |  |  | **1** |  |  |  |  |  |
| **NADP-dependent malic enzyme** | **P48163** | **1** |  |  |  |  | **1** |  |  |  |  |  |  |  |
| **NADH dehydrogenase [ubiquinone] 1 alpha subcomplex subunit 13** | **Q9P0J0** | **1** |  |  |  |  | **1** |  |  |  |  |  |  |  |
| **NADH dehydrogenase [ubiquinone] 1 beta subcomplex subunit 9** | **Q9Y6M9** | **1** |  |  |  |  | **1** |  |  |  |  |  |  |  |
| **NADH dehydrogenase [ubiquinone] 1 beta subcomplex subunit 2, mitochondrial** | **O95178** | **1** |  |  |  |  | **1** |  |  |  |  |  |  |  |
| **NADH dehydrogenase [ubiquinone] 1 beta subcomplex subunit 10** | **O96000** | **1** |  |  | **1** |  |  |  |  |  |  |  |  |  |
| **NADH dehydrogenase [ubiquinone] flavoprotein 1, mitochondrial** | **P49821** | **1** |  |  |  |  |  |  |  |  |  | **1** |  |  |
| **NEDD8** | **Q15843** | **1** |  |  |  |  | **1** |  |  |  |  |  |  |  |
| **Mitochondrial NFS1 nitrogen fixation 1** | **Q53FP3** | **1** |  | **1** |  |  |  |  |  |  |  |  |  |  |
| **Neurobeachin-like protein 1** | **Q6ZS30** | **1** |  |  |  |  | **1** |  |  |  |  |  |  |  |
| **Neuroblastoma-amplified sequence** | **A2RRP1** | **1** |  | **1** |  |  |  |  |  |  |  |  |  |  |
| **Neuronal growth regulator 1** | **Q7Z3B1** | **1** |  |  |  |  | **1** |  |  |  |  |  |  |  |
| **N(G),N(G)-dimethylarginine dimethylaminohydrolase 2** | **O95865** | **1** |  |  |  |  | **1** |  |  |  |  |  |  |  |
| **Neurosecretory protein VGF** | **O15240** | **1** |  |  |  |  | **1** |  |  |  |  |  |  |  |
| **Nicotinamide mononucleotide adenylyltransferase 3** | **Q96T66** | **1** |  | **1** |  |  |  |  |  |  |  |  |  |  |
| **Nitrilase homolog 1** | **Q86X76** | **1** |  |  |  |  | **1** |  |  |  |  |  |  |  |
| **Non-SMC element 4 homolog A** | **Q9NXX6** | **1** |  |  |  |  | **1** |  |  |  |  |  |  |  |
| **Non-histone chromosomal protein HMG-17** | **P05204** | **1** |  |  |  |  | **1** |  |  |  |  |  |  |  |
| **Nucleosome assembly protein 1-like 1** | **P55209** | **1** |  |  |  |  | **1** |  |  |  |  |  |  |  |
| **Numb-like protein** | **Q9Y6R0** | **1** |  |  |  |  | **1** |  |  |  |  |  |  |  |
| **Olfactory receptor 8K3** | **Q8NH51** | **1** |  |  |  |  | **1** |  |  |  |  |  |  |  |
| **Palladin** | **Q8WX93** | **1** |  |  |  |  | **1** |  |  |  |  |  |  |  |
| **Peptidyl Prolyl Peptidyl-prolyl cis-trans isomerase NIMA-interacting 1 Cis/Trans-Isomerase** | **Q13526** | **1** |  |  |  |  |  |  |  | **1** |  |  |  |  |
| **Peptidyl-prolyl cis-trans isomerase FKBP1A** | **P62942** | **1** |  |  |  |  | **1** |  |  |  |  |  |  |  |
| **Phosphatidylinositol-binding clathrin assembly protein** | **Q13492** | **1** |  |  |  |  | **1** |  |  |  |  |  |  |  |
| **Phosphatidylinositol 3,4,5-trisphosphate-dependent Rac exchanger 1 protein** | **Q8TCU6** | **1** |  | **1** |  |  |  |  |  |  |  |  |  |  |
| **Phosphatidylinositol transfer protein alpha isoform** | **Q00169** | **1** |  |  |  |  | **1** |  |  |  |  |  |  |  |
| **Phosphoglycolate phosphatase** | **A6NDG6** | **1** |  |  |  |  | **1** |  |  |  |  |  |  |  |
| **Phospholipid-transporting ATPase IA** | **Q9Y2Q0** | **1** |  | **1** |  |  |  |  |  |  |  |  |  |  |
| **Phytanoyl-CoA dioxygenase domain-containing protein 1** | **Q5SRE7** | **1** |  | **1** |  |  |  |  |  |  |  |  |  |  |
| **Plectin** | **Q15149** | **1** |  |  |  |  |  |  |  |  |  | **1** |  |  |
| **plectin-1** | **Q15149-2** | **1** |  |  |  |  |  | **1** |  |  |  |  |  |  |
| **Polypyrimidine tract-binding protein 2** | **Q9UKA9** | **1** |  |  |  |  | **1** |  |  |  |  |  |  |  |
| **Prefoldin subunit 3** | **P61758** | **1** |  |  |  |  | **1** |  |  |  |  |  |  |  |
| **Prenylcysteine oxidase-like** | **Q8NBM8** | **1** |  |  |  |  | **1** |  |  |  |  |  |  |  |
| **Probable ATP-dependent RNA helicase DDX46** | **Q7L014** | **1** |  | **1** |  |  |  |  |  |  |  |  |  |  |
| **Profilin-2** | **P35080** | **1** |  |  |  |  | **1** |  |  |  |  |  |  |  |
| **Prohibitin** | **P35232** | **1** |  |  |  |  |  |  |  |  |  | **1** |  |  |
| **Prolyl endopeptidase** | **P48147** | **1** | **1** |  |  |  |  |  |  |  |  |  |  |  |
| **Protein AHNAK2** | **Q8IVF2** | **1** |  |  |  |  | **1** |  |  |  |  |  |  |  |
| **Protein bassoon** | **Q9UPA5** | **1** |  |  |  |  | **1** |  |  |  |  |  |  |  |
| **Protein FAM49A** | **Q9H0Q0** | **1** |  |  |  |  | **1** |  |  |  |  |  |  |  |
| **Protein S100-A13** | **Q99584** | **1** |  |  |  |  | **1** |  |  |  |  |  |  |  |
| **Protein kinase C alpha type** | **P17252** | **1** |  |  |  |  | **1** |  |  |  |  |  |  |  |
| **Protein kinase C theta type** | **Q04759** | **1** |  |  |  |  | **1** |  |  |  |  |  |  |  |
| **Protein kinase C gamma type** | **P05129** | **1** |  |  |  |  | **1** |  |  |  |  |  |  |  |
| **Protein phosphatase PTC7 homolog** | **Q8NI37** | **1** |  | **1** |  |  |  |  |  |  |  |  |  |  |
| **Protein Wnt-10a** | **Q9GZT5** | **1** |  |  |  |  | **1** |  |  |  |  |  |  |  |
| **proteasome subunit alpha type-1** | **P25786** | **1** |  |  |  |  |  | **1** |  |  |  |  |  |  |
| **Proto-oncogene tyrosine-protein kinase Yes** | **P07947** | **1** |  |  |  |  | **1** |  |  |  |  |  |  |  |
| **Proto-oncogene tyrosine-protein kinase Fyn** | **P06241** | **1** |  |  |  |  | **1** |  |  |  |  |  |  |  |
| **Putative hexokinase HKDC1** | **Q2TB90** | **1** |  |  |  |  | **1** |  |  |  |  |  |  |  |
| **Ras GTPase-activating-like protein IQGAP1** | **P46940** | **1** |  |  |  |  | **1** |  |  |  |  |  |  |  |
| **Ras-related protein Rap-2a** | **P10114** | **1** |  |  |  |  | **1** |  |  |  |  |  |  |  |
| **Ras-related protein Rap-2b** | **P61225** | **1** |  |  |  |  | **1** |  |  |  |  |  |  |  |
| **Ran-binding protein 6** | **O60518** | **1** |  |  |  |  | **1** |  |  |  |  |  |  |  |
| **Regulating synaptic membrane exocytosis protein 1** | **Q86UR5** | **1** |  | **1** |  |  |  |  |  |  |  |  |  |  |
| **Regulator of microtubule dynamics protein 3** | **Q96TC7** | **1** |  |  |  |  | **1** |  |  |  |  |  |  |  |
| **RILP-like protein 1** | **Q5EBL4** | **1** |  |  |  |  | **1** |  |  |  |  |  |  |  |
| **Septin-4** | **O43236** | **1** |  |  |  |  |  |  | **1** |  |  |  |  |  |
| **Serine/threonine-protein kinase SMG1** | **Q96Q15** | **1** |  |  |  |  |  |  | **1** |  |  |  |  |  |
| **Serine/threonine-protein kinase MRCK alpha** | **Q5VT25** | **1** |  |  |  |  | **1** |  |  |  |  |  |  |  |
| **Serine/threonine-protein phosphatase 2A 56 kDa regulatory subunit epsilon isoform** | **Q16537** | **1** |  |  |  |  | **1** |  |  |  |  |  |  |  |
| **Serine/threonine-protein phosphatase 2A regulatory subunit B'** | **Q15257** | **1** |  |  |  |  | **1** |  |  |  |  |  |  |  |
| **Serine/threonine-protein phosphatase 2A 55 kDa regulatory subunit B delta isoform** | **Q66LE6** | **1** |  |  |  |  | **1** |  |  |  |  |  |  |  |
| **Serine/threonine-protein phosphatase PP1-beta catalytic subunit** | **P62140** | **1** |  |  |  |  | **1** |  |  |  |  |  |  |  |
| **Serum albumin** | **P02768** | **1** |  |  |  |  |  |  |  |  |  | **1** |  |  |
| **Sideroflexin-1** | **Q9H9B4** | **1** |  |  |  |  |  |  |  |  |  | **1** |  |  |
| **Sideroflexin-5** | **Q8TD22** | **1** |  |  |  |  | **1** |  |  |  |  |  |  |  |
| **Splicing factor U2AF 65 kDa subunit** | **P26368** | **1** |  |  |  |  | **1** |  |  |  |  |  |  |  |
| **Sodium channel protein type 1 subunit alpha** | **P35498** | **1** |  |  |  |  | **1** |  |  |  |  |  |  |  |
| **Sodium/potassium-transporting ATPase subunit beta-2** | **P14415** | **1** |  |  |  |  | **1** |  |  |  |  |  |  |  |
| **Sorbin and SH3 domain-containing protein 2** | **O94875** | **1** |  |  |  |  | **1** |  |  |  |  |  |  |  |
| **Sorting nexin-2** | **O60749** | **1** |  |  |  |  | **1** |  |  |  |  |  |  |  |
| **Sulfotransferase 4A1** | **Q9BR01** | **1** |  |  |  |  | **1** |  |  |  |  |  |  |  |
| **Superoxide dismutase** | **P00441** | **1** |  |  |  |  |  |  |  |  |  | **1** |  |  |
| **Synaptopodin** | **Q8N3V7** | **1** |  |  |  |  | **1** |  |  |  |  |  |  |  |
| **Synaptogyrin 3** | **O43761** | **1** |  |  |  | **1** |  |  |  |  |  |  |  |  |
| **Syntaxin-binding protein 5** | **Q5T5C0** | **1** |  | **1** |  |  |  |  |  |  |  |  |  |  |
| **T-lymphocyte activation antigen CD86** | **P42081** | **1** |  |  |  |  | **1** |  |  |  |  |  |  |  |
| **tau tubulin kinase1** | **Q5TCY1** | **1** |  |  |  | **1** |  |  |  |  |  |  |  |  |
| **THAP domain-containing protein 2** | **Q9H0W7** | **1** |  |  |  |  | **1** |  |  |  |  |  |  |  |
| **Thy-1 membrane glycoprotein** | **P04216** | **1** |  |  |  |  |  |  |  |  |  | **1** |  |  |
| **Thyroid adenoma-associated protein** | **Q6YHU6** | **1** |  |  |  |  | **1** |  |  |  |  |  |  |  |
| **Transgelin-2** | **P37802** | **1** |  |  |  |  | **1** |  |  |  |  |  |  |  |
| **Translin** | **Q15631** | **1** |  |  |  |  | **1** |  |  |  |  |  |  |  |
| **Transmembrane protein 126A** | **Q9H061** | **1** |  |  |  |  | **1** |  |  |  |  |  |  |  |
| **Trichohyalin** | **Q07283** | **1** |  |  |  |  | **1** |  |  |  |  |  |  |  |
| **Tripartite motif-containing protein 3** | **O75382** | **1** |  |  |  |  | **1** |  |  |  |  |  |  |  |
| **Tropomodulin-2** | **Q9NZR1** | **1** |  |  |  |  | **1** |  |  |  |  |  |  |  |
| **Tropomyosin alpha-3 chain** | **P06753** | **1** |  |  |  |  | **1** |  |  |  |  |  |  |  |
| **Tropomyosin alpha-4 chain** | **P67936** | **1** |  |  |  |  | **1** |  |  |  |  |  |  |  |
| **Tyrosine-protein phosphatase non-receptor type substrate 1** | **P78324** | **1** |  |  |  |  | **1** |  |  |  |  |  |  |  |
| **Tyrosine-protein phosphatase non-receptor type 23** | **Q9H3S7** | **1** |  |  |  |  | **1** |  |  |  |  |  |  |  |
| **Tryptophanyl-tRNA synthetase, mitochondrial** | **Q9UGM6** | **1** |  |  |  |  | **1** |  |  |  |  |  |  |  |
| **U1 small nuclear ribonucleoprotein 70 kDa** | **P08621** | **1** |  | **1** |  |  |  |  |  |  |  |  |  |  |
| **U1 small nuclear ribonucleoprotein A** | **P09012** | **1** |  | **1** |  |  |  |  |  |  |  |  |  |  |
| **UPF0557 protein C10orf119** | **Q9BTE3** | **1** |  |  |  |  | **1** |  |  |  |  |  |  |  |
| **Undifferentiated embryonic cell transcription factor 1** | **Q5T230** | **1** |  |  |  |  | **1** |  |  |  |  |  |  |  |
| **Ubiquitin-like modifier-activating enzyme 6** | **A0AVT1** | **1** |  |  |  |  | **1** |  |  |  |  |  |  |  |
| **UDP-glucose 6-dehydrogenase** | **O60701** | **1** |  |  |  |  | **1** |  |  |  |  |  |  |  |
| **UPF0631 protein HSD24** | **A8MSI8** | **1** |  |  |  |  | **1** |  |  |  |  |  |  |  |
| **Vacuolar protein sorting-associated protein 29** | **Q9UBQ0** | **1** |  |  |  |  | **1** |  |  |  |  |  |  |  |
| **Vacuolar protein sorting-associated protein 26B** | **Q4G0F5** | **1** |  |  |  |  | **1** |  |  |  |  |  |  |  |
| **Vasorin** | **Q6EMK4** | **1** |  |  |  |  | **1** |  |  |  |  |  |  |  |
| **Very long-chain specific acyl-CoA dehydrogenase** | **P49748** | **1** | **1** |  |  |  |  |  |  |  |  |  |  |  |
| **V-type proton ATPase subunit d 1** | **P61421** | **1** |  |  |  |  |  |  |  |  |  | **1** |  |  |
| **V-type proton ATPase subunit F** | **Q16864** | **1** | **1** |  |  |  |  |  |  |  |  |  |  |  |
| **Vacuolar proton translocating ATPase 116 kDa subunit** | **Q93050** | **1** |  |  |  |  |  |  |  |  |  | **1** |  |  |
| **Voltage-dependent anion-selective channel protein 2** | **P45880** | **1** |  |  |  |  |  |  |  |  |  | **1** |  |  |
| **Voltage-dependent anion-selective channel protein 3** | **Q9Y277** | **1** |  |  |  |  |  |  |  |  |  | **1** |  |  |
| **Protein NipSnap homolog 2** | **O75323** | **1** | **1** |  |  |  |  |  |  |  |  |  |  |  |
| **WD repeat and FYVE domain-containing protein 3** | **Q8IZQ1** | **1** |  | **1** |  |  |  |  |  |  |  |  |  |  |
| **WD repeat-containing protein 61** | **Q9GZS3** | **1** |  |  |  |  | **1** |  |  |  |  |  |  |  |
| **zinc finger protein 430** | **Q9H8G1** | **1** |  |  |  | **1** |  |  |  |  |  |  |  |  |
|  |  |  |  |  |  |  |  |  |  |  |  |  |  |  |
|  |  |  |  |  |  |  |  |  |  |  |  |  |  |  |
