# Supplementary material for: Alzheimer's disease: are blood and brain markers related? A systematic review
Source: Ann Clin Transl Neurol. 2016 May 11;3(6):455–62. doi: 10.1002/acn3.313 (PMC4891999; doi:10.1002/acn3.313)
Supplement: Supplementary file 3 — Table S3. Proteins identified as being differentially expressed from blood studies of Alzheimer's disease (AD) patients and brain studies of AD patient. [file ACN3-3-455-s003.docx]

Supplementary Table 3 Proteins identified as being differentially expressed from blood studies of Alzheimer’s disease (AD) patients and brain studies of AD patient

| Protein | UniProt ID | Brain studies | Blood studies |
| --- | --- | --- | --- |
| 14-3-3 protein epsilon | P62258 | **3** ^13, 16, 17^ | **1** ^43^ |
| Glyceraldehyde-3-phosphate dehydrogenase | P04406 | **3** ^13, 19, 20^ | **1** ^43^ |
| Calcium/calmodulin-dependent protein kinase type II subunit alpha | Q9UQM7 | **2** ^13, 22^ | **1** ^40^ |
| Complement C4-A | P0C0L4 | **2** ^14, 17^ | **3** ^30, 32, 36^ |
| Gelsolin | P06396 | **2** ^17, 22^ | **1** ^42^ |
| Peroxiredoxin-2 | P32119 | **1** ^20^ | **1** ^43^ |
| Actin, cytoplasmic 1 | P60709 | **1** ^13^ | **1** ^43^ |
| Apolipoprotein E | P02649 | **1** ^14^ | **6** ^25, 26, 27, 28, 29, 30^ |
| Hemoglobin subunit alpha | P69905 | **1** ^13^ | **1** ^30^ |
| Histidine triad nucleotide-binding protein 1 | P49773 | **1** ^17^ | **1** ^41^ |
| alpha-2-macroglobulin | P01023 | **1** ^18^ | **6** ^30, 31, 32, 33, 34, 35^ |
| Complement component C3 | P01024 | **1** ^14^ | **5** ^26, 28, 30, 34, 36^ |
| Serum albumin | P02768 | **1** ^22^ | **4** ^27, 28, 32, 36^ |
| Complement C4-B | P0C0L5 | **1** ^14^ | **2** ^30, 32^ |
| Ig alpha-1 chain C region | P01876 | **1** ^13^ | **1** ^32^ |
| Ig gamma-1 chain C region | P01857 | **1** ^13^ | **1** ^32^ |
| Superoxide dismutase | P00441 | **1** ^22^ | **1** ^27^ |
| Annexin A1 | P04083 | **1** ^17^ | **1** ^43^ |
